# Gene and protein nomenclature in public databases

**DOI:** 10.1186/1471-2105-7-372

**Published:** 2006-08-09

**Authors:** Katrin Fundel, Ralf Zimmer

**Affiliations:** 1 Institut für Informatik, Ludwig-Maximilians-Universität München, Amalienstrasse 17, 80333 München, Germany

## Abstract

**Background:**

Frequently, several alternative names are in use for biological objects such as genes and proteins. Applications like manual literature search, automated text-mining, named entity identification, gene/protein annotation, and linking of knowledge from different information sources require the knowledge of all used names referring to a given gene or protein. Various organism-specific or general public databases aim at organizing knowledge about genes and proteins. These databases can be used for deriving gene and protein name dictionaries. So far, little is known about the differences between databases in terms of size, ambiguities and overlap.

**Results:**

We compiled five gene and protein name dictionaries for each of the five model organisms (yeast, fly, mouse, rat, and human) from different organism-specific and general public databases. We analyzed the degree of ambiguity of gene and protein names within and between dictionaries, to a lexicon of common English words and domain-related non-gene terms, and we compared different data sources in terms of size of extracted dictionaries and overlap of synonyms between those.

The study shows that the number of genes/proteins and synonyms covered in individual databases varies significantly for a given organism, and that the degree of ambiguity of synonyms varies significantly between different organisms. Furthermore, it shows that, despite considerable efforts of co-curation, the overlap of synonyms in different data sources is rather moderate and that the degree of ambiguity of gene names with common English words and domain-related non-gene terms varies depending on the considered organism.

**Conclusion:**

In conclusion, these results indicate that the combination of data contained in different databases allows the generation of gene and protein name dictionaries that contain significantly more used names than dictionaries obtained from individual data sources. Furthermore, curation of combined dictionaries considerably increases size and decreases ambiguity.

The entries of the curated synonym dictionary are available for manual querying, editing, and PubMed- or Google-search via the ProThesaurus-wiki. For automated querying via custom software, we offer a web service and an exemplary client application.

## Background

Genes and proteins are biological objects of primary importance for understanding biochemical processes. The exchange of knowledge on any kind of object requires consistent names or identifiers for each object. So far, even though nomenclature paradigms are provided by several communities, the generation and assignment of names to newly identified genes and proteins is not strictly standardized and standards are not strictly enforced; i.e. every researcher is free to define, assign and use names as required in particular in scientific papers. Thus, most genes/proteins are referred to by several names (synonymy), and a name can be associated with several genes/proteins (homonymy) which causes ambiguity. Furthermore, gene symbols and names can overlap with English words, such as the gene names *leg*, *white*, and *key*.

Public databases aim at organizing information by assigning unique identifiers to genes and proteins. Besides sequences, biochemical properties, and other information, these databases also contain gene and protein names. These databases are quite diverse in terms of organism-specificity, structure, and applied curation procedure. For well-studied organisms several databases can be consulted from organism specific databases like the Saccharomyces Genome Database [1], FlyBase [2], Mouse Genome Informatics [3], Rat Genome Database [4], and HUGO [5], to general gene databases like Entrez Gene [6] (formerly LocusLink) and the manually curated gene and protein collection Swiss-Prot [7]. The latter contains high-quality annotations, yet the protein names are contained in different database fields: the "Gene name" field, which is easy to parse automatically and the description field, that contains long forms, and is more difficult to parse due to nested parentheses which sometimes represent separate synonyms of varying specificity and sometimes are subtypes, specifications of or additions to previous synonyms (e.g.: "(Na(+)/I(-)-symporter)" or "Amyloid beta A4 protein precursor (APP) (ABPP) (Alzheimer's disease amyloid protein homolog) [Contains: Soluble APP-alpha (S-APP-alpha); C99; Beta-amyloid protein 42 (Beta-APP42); C83; P3(42); P3(40); Gamma-CTF(59) (Gamma-secretase C-terminal fragment 59); C31]."). The usage of all information contained in this database thus comes with considerable effort but increases size of the resulting protein name lists.

Benchmarking of gene and protein name lists is an important issue. Currently, there is no gold-standard for estimating coverage and ambiguity or recall and precision for gene or protein name lists. In order to accomplish this, one would need to gather all names used in any data source (database or free text) for a given gene or protein and assign these to unique object identifiers. The BioCreAtIvE challenge can be considered as a small-scale benchmark for gene and protein name lists in that the participants were required to recover numerous gene names from text and return unique identifiers (Task 1B). Our results in the BioCreAtIvE assessment [8,9] have shown that dictionaries compiled from public databases are very useful for identifying gene/protein names; even with simple matching strategies they allow high recall and precision when carefully curated.

Many systems aiming at the identification of gene and protein names [10-13] make use of the names and aliases from public databases. A frequent approach consists in the compilation of large dictionaries of gene names that are subsequently used for matching text fragments to database identifiers. Yet, so far, little is known about the relevance of the choice of the database to use on the results in terms of size and ambiguity.

The work presented in this paper focuses on the analysis of gene and protein names and gene name dictionaries in terms of the following features: (1) Ambiguity of gene names, with respect to different gene objects within a species and with respect to gene objects of different species. The intra-species analysis is done for each data source separately and for joined dictionaries from different data sources for each species. The inter-species analysis is done for the combined dictionaries. (2) Degree of overlap of gene names for a given species in different public databases. This indicates the relevance and the possible gain of joining information from different data sources. (3) Ambiguity of the combined dictionaries with general English words and with non-gene and non-protein, but domain related terms. This indicates the difficulty of accurate text searches and hence the effort that has to be spent on contextual filtering. (4) Ambiguity of gene names dictionaries after extensive curation; this indicates the relevance and effectiveness of curation for the given dictionary and the residual necessity of contextual filtering.

Genes and the derived proteins usually carry the same name. The databases analyzed here except for Swiss-Prot are focused on genes rather than proteins. In this study, we do not distinguish between gene and protein names and thus use the terms genes and proteins as synonyms.

In this work, a number of optimized synonym lists have been derived, which are helpful for various purposes. These lists need to be maintained; new data need to be integrated, errors need to be detected and removed, and new objects and synonyms need to be added to the dictionary. A wiki [14] allows users to edit and comment entries in a standardized, comfortable and well-known way (see also: [15]).

We provide the entries of the curated synonym lists derived in this paper via the ProThesaurus-wiki [16]. The curation of the information maintained in the wiki together with the automated generation procedure needs to be done regularly. For automated querying via custom software, we offer a web service and an exemplary client application [17].

### Related work

Several studies dealing with ambiguity in biomedical nomenclatures have been accomplished during the last few years. Most of these focus on the analysis, i.e. detection and mapping, of abbreviations [18-21], or on the compilation of databases containing mappings between abbreviations and the corresponding terms [22,23]. This is due to the omnipresence of abbreviations in the biomedical domain and to the significant problems they entail. Abbreviations frequently have numerous different meanings, which can belong to the same or distinct semantic fields, e.g. protein names, experimental techniques, cell lines, or others. Furthermore, authors frequently define their own abbreviations and names, which are then more or less only valid for the document they are contained in and possibly closely related documents.

Another focus of studies is disambiguation, which concentrates on identifying the correct meaning of an expression out of a set of possible alternatives. This concerns abbreviations as well as words or even longer expressions [24,25].

A study on the ambiguity of human gene symbols [26] showed that gene symbols from LocusLink overlap with abbreviations and that many of the corresponding occurrences in MEDLINE abstracts are not related to the corresponding gene. Another study of Hirschman *et al*. [27] investigated the problems encountered when identifying biological names in texts, this study describes the challenge of recognizing fly gene names in detail.

BioCreAtIvE [28] was a first independent assessment for methods aiming at gene name identification in texts, which became significant not only because of the evaluation of the methods applied by the participants, but also because of its setup: The task was to identify gene names from three frequently used model organisms (yeast, mouse, and fly), and thereby the results impressively demonstrate the difference in performance various methods achieved for the different organisms. For yeast, most groups achieved F-measures (harmonic mean of precision and recall) between 80% and 90%, while for mouse and fly, results were wide-spread and only a few groups achieved F-measures about 80%, which clearly reflects the varying difficulty of the corresponding gene nomenclatures.

The first study known to us aiming at a systematic comparison of gene nomenclatures from different organisms [29] analyzed the nomenclatures of mouse, fly, worm, and yeast. The authors evaluated ambiguity within and across nomenclatures and with general English by exact matching of symbols and names, and applied an NLP (natural language processing) system for analyzing recall and ambiguity of matching the derived mouse dictionary against a set of MEDLINE abstracts. Eukaryotic gene name ambiguity was analyzed in terms of intra-species ambiguity, ambiguity with general English and medical terms and across species by Chen *et al*. [30]. This work focused on the comparison of a large number of organisms with regard to differences in ambiguity between official gene symbols and aliases, and they analyzed author preferences for symbols or full names.

The work presented here extends the previous ones by analyzing ambiguity within and between dictionaries, (1) by using three different definitions for term equivalence, which reveals some properties of the analyzed nomenclatures; (2) by evaluating different public data sources for extracting dictionaries separately, which allows an individual rating of the different data sources; and (3) by analyzing the degree of overlap of gene names contained in different data sources, which clearly demonstrates the necessity of combining information from multiple data sources when aiming at generation of complete gene name dictionaries.

## Results and discussion

### Size of gene name dictionaries

**Figure 1:**
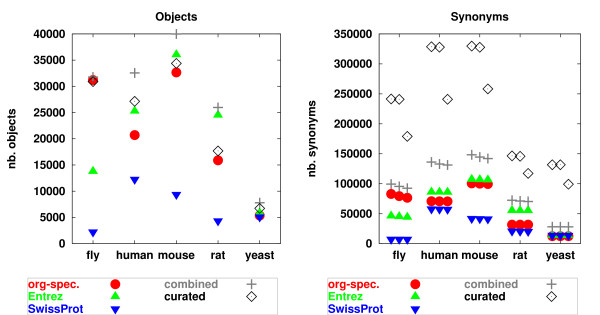
**Size of gene name dictionaries**. Number of objects (left plot) and synonyms (right plot) for gene name dictionaries compiled from different data sources (organism-specific database: Yeast: Saccharomyces Genome Database, Fly: FlyBase, Mouse: Mouse Genome Informatics, Rat: Rat Genome Database, Human: HUGO; 'combined' is the merged dictionary from the organism-specific database, Swiss-Prot and Entrez Gene; 'curated' is additionally expanded and pruned). In the right plot, the three marks for each dictionary correspond to the three definitions of equivalence: exact, mixed, and normalized, respectively, from left to right. For details see section 'Compilation of gene name dictionaries'.

The sizes of gene name dictionaries obtained from different data sources are shown in Figure 1. The figure shows that the dictionaries vary significantly in their size, between different organisms as well as between different data sources. Interestingly, the number of entries in the different databases for a given organism varies significantly, e.g. there is a factor of approx. 15 between the number of objects for *Drosophila *in FlyBase compared to Swiss-Prot. One reason for the large number of entries in FlyBase is that this database also includes genes that have been introduced into *Drosophila *(transgenes).

There is no general tendency to whether organism-specific or one of the general databases contain more entries. In part, the variance between databases might be explained by their different scope and objectives, e.g. the data in Swiss-Prot is manually curated and this is a reason for the smaller number of objects.

It is important to note that the difficulty of extracting relevant gene names from the data files varies significantly between the different data sources. Most files have a format that is easy to parse, with defined separators between distinct names. Some databases use individual conventions for representing special characters, greek letters or formatting of name parts (e.g. "&bgr;'-Cop" for "beta'-Cop" in FlyBase, or "Cypllb2<sup>ml</sup>" for "Cypllb2^ml^" in RGD). In order to generate dictionaries applicable for named entity recognition systems, these formatting conventions need to be accounted for. Swiss-Prot is the database which is most difficult to parse among the databases analyzed here. This is due to the choice of parentheses as separators between long names. Given the fact that long names frequently contain parentheses, this entails the necessity of a more involved parser than necessary for other data sources. Furthermore, protein long names are contained in the description section, which also contains further information, e.g. when a protein has several functional domains which have individual names, this is specified by an expression of the format "entire_protein [Includes: domain_l_name; domain_2_name]".

Figure 1 also shows that the curation procedure leads to a modest decrease in the number of objects, which is due to merging of objects that have a significant number of synonyms in common, and to a significant increase in the number of synonyms, which is mainly due to the addition of spelling variants.

The entries of the curated dictionaries are available via our ProThesaurus-wiki [16] and web service [17].

### Intra-species ambiguity

**Figure 2:**
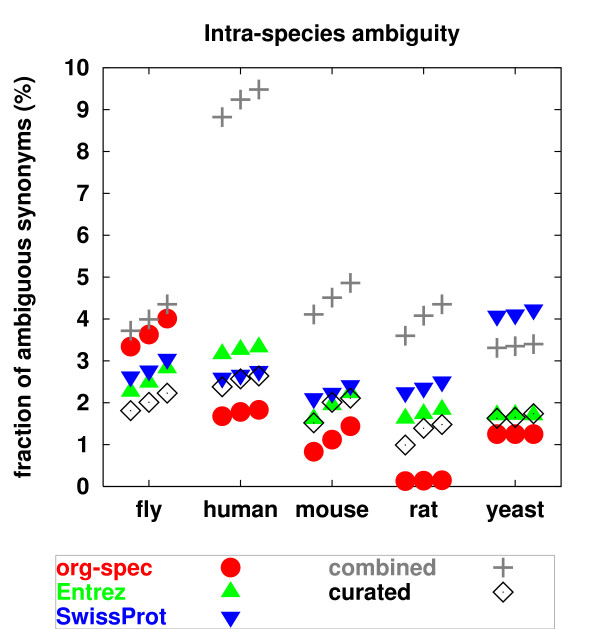
**Ambiguity within gene name dictionaries**. The ambiguity within gene name dictionaries derived from different data sources and for different organisms varies significantly. Combined dictionaries generally show relatively high ambiguity, curation reduces ambiguity. For notation see Figure 1, for details see section 'Intra-species Ambiguity'.

Figure 2 shows the degree of intra-species ambiguity, i.e. the fraction of synonyms that are assigned to more than one object within a gene name dictionary. The figure shows that the degree of ambiguity varies significantly between different organisms. When considering the combined dictionaries, yeast shows the lowest number of ambiguous synonyms and human shows the highest number. Previous studies [29,30] investigated the intra-species ambiguity of dictionaries combined from organism-specific databases and LocusLink (now Entrez Gene). Our results agree with their findings, only for fly they obtained significantly higher ambiguity (>12%) than we do (1.8–4.4%). This might be due to the fact that we restrict entries from FlyBase to those specifically assigned to *Drosophila melanogaster*.

Interestingly, the degree of ambiguity (*DoA*) of the individual dictionaries for a given organism varies significantly between the different data sources, e.g. the degree of ambiguity for the human dictionary derived from HUGO is 1.68%–1.83%. The *DoA *of the human Entrez Gene dictionary is 3.16%–3.32%, even though the number of synonyms is similar for the two dictionaries.

This figure also shows the influence of different definitions of term equivalence; e.g. for yeast the difference between the three measures is very small, while for all other organisms it is significantly larger. This indicates that for yeast, slight spelling variances generally do not harm, while for other organisms case and exact spelling can distinguish between one gene or another.

Furthermore, the results show whether the combination of individual dictionaries lead to an increase in ambiguity. For yeast and fly, the degree of ambiguity of the combined dictionary corresponds to the highest degree of ambiguity of the individual dictionaries. For human, mouse, and rat the ambiguities of the combined dictionaries are significantly higher than the ambiguities of the individual dictionaries. This is most pronounced for human; here the individual dictionaries show a *DoA *of 1.7–3.3% and the combined dictionary shows a *DoA *of 8.8–9.5%. This presumably indicates that entries in the different databases correspond to each other, even though they are not mapped to each other in the mappings derived from the considered databases. Thus, the mappings between databases are presumably deficient.

For all organisms, the curation procedure leads to a significant reduction of ambiguous terms; this is due to the removal of unspecific synonyms, and to the merging of objects sharing a large number of equivalent synonyms. The *DoA *of the considered dictionaries after curation is 1–2.6%.

### Inter-species ambiguity

The degree of inter-species ambiguity is shown in Table 1. The table shows that there is a significant variability in ambiguity between different species. Yeast and fly generally have a very low degree of ambiguity with other organisms, while the ambiguity between mouse, rat, and human is significantly higher.

This can be explained by the fact that mouse, rat, and human are much closer related to each other than to yeast, and homologs in different organisms often carry the same name [30]. For the mammals in our test set this explains a significant part of the ambiguity of synonyms. The highest degree of ambiguity is found between human and mouse, ranging between 15% and 25% for the different measures. The nomenclature guidelines from MGD and RGD explicitely state that 'genes that are recognizable orthologs of already-named human genes should be given the same name and symbol as the human gene'; and also the HUGO guideline states 'that homologous genes in different vertebrate species should where possible have the same gene nomenclature' and that 'the agreement between human and mouse gene nomenclature for many homologuous genes should be continued and extended to other vertebrate species where possible'. Generally, the nomenclatures of rat, human, and mouse genes are coordinated with each other by the corresponding committees. This brings about mappings between orthologs by cross-references, co-assignment of nomenclatures to ortholog genes and thus an increasing unification of the individual nomenclatures.

The curation has diverse effects on the inter-species ambiguity: while the comparisons of the dictionaries of human, mouse, and rat show higher inter-species ambiguity for curated dictionaries than for combined dictionaries, the comparisons of dictionaries of other pairs of organisms show lower inter-species ambiguity for curated dictionaries. An increase in inter-species ambiguity is due to the expansion of synonyms, e.g. the expansion of abbreviations, which can result in equivalent synonyms that were not present originally in the two lists to be compared. A decrease in inter-species ambiguity can be explained by the removal of unspecific synonyms, but also by the increase in total number of synonyms emerging from the expansion of abbreviations and addition of spelling variants.

### Overlap between different data sources

**Figure 3:**
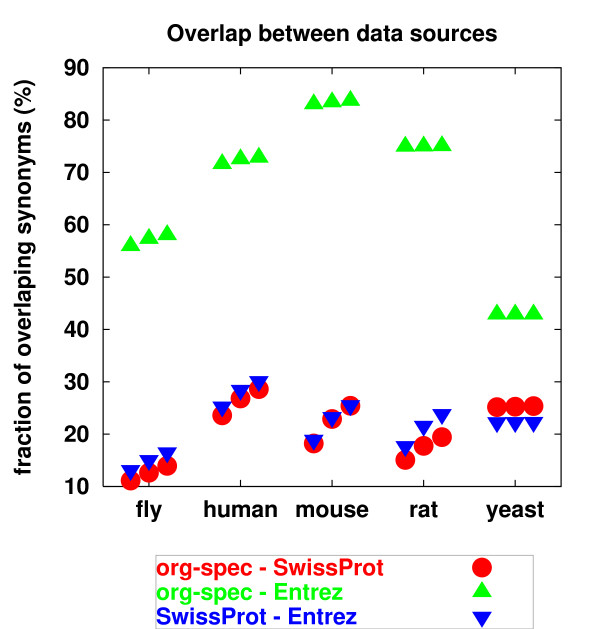
**Overlap between different data sources**. The overlap between gene name dictionaries compiled from different data sources varies for different organisms and pairs of databases. Organism-specific databases and Entrez Gene show highest overlap for all organisms. For notation see Figure 1, for details see section 'Overlap between different data sources'.

The degree of overlap of synonyms between different data sources is shown in Figure 3. The figure shows that the overlap in synonyms between the investigated data sources varies between 11% and 83%. Particularly, the overlap between the two general data sources Swiss-Prot and Entrez Gene as well as between Swiss-Prot and organism-specific databases is relatively low. The overlap between organism-specific databases and Entrez Gene is significantly higher than the overlap between the other pairs of databases.

These results strengthen the hypothesis that it is necessary to combine entries from several data sources in order to obtain a dictionary that is as complete as possible.

The results once again vary significantly when the different definitions of equivalence are applied. For the overlap between organism specific databases and Entrez Gene the difference between the different measures of equivalence is quite small, indicating that the gene names in these databases are more or less identical. For the comparison between organism-specific databases and Swiss-Prot there are some important differences, e.g. for mouse, the overlap is only 18% when exact identity is required, but 25% when gene names are normalized. This signifies that numerous gene names in these databases are not exactly identical, but their normalized forms are the same and thus they are very similar.

The differences in overlap are presumably due to the structures and strategies of the organizations that maintain the databases. The organizations maintaining the organism-specific databases are the authorities for official nomenclature and genome annotation. Model organism databases and general sequence repository resources like NCBI Entrez Gene exchange data on a regular basis to reflect the official nomenclature. Swiss-Prot also works together with model organism databases. Entrez Gene and Swiss-Prot are historically separated as they differ in their focus. NCBI established Entrez Gene as a database for gene-specific information, it focuses on genomes that have been completely sequenced or that have an active research community to contribute gene-specific information. Swiss-Prot is an annotated protein sequence database; the annotation is done manually and concerns, besides nomenclature, protein structure, function, associated diseases. The UniProt consortium is concerned with integrating information in the UniProt Knowledge-base. This provides a central, stable, comprehensive, fully classified, richly and accurately annotated protein sequence database with extensive cross-references to other data sources. Currently, Swiss-Prot forms part of the UniProt Knowledgebase. With the advent of the UniProt project, the expectation will be that Swiss-Prot/UniProt and Entrez Gene will increasingly share nomenclature and that the mapping between databases will be increasingly complete and unambiguous. This will facilitate the generation of gene name dictionaries and text mining applications.

### Ambiguity with English lexicon and domain-related terms

**Figure 4:**
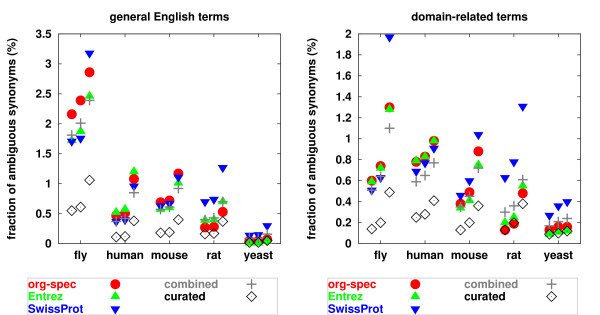
**Ambiguity between gene name dictionaries and general English terms and domain-related terms**. Ambiguity between gene name dictionaries and general English terms (left plot) and domain-related non-gene and non-protein terms (right plot). Fly shows highest ambiguity with general English terms. All dictionaries show higher ambiguity for normalized gene names than for exact gene names. For notation see Figure 1, for details see section 'Ambiguity with English lexicon and domain-related terms'.

Figure 4 shows the degree of ambiguity between the dictionaries and a lexicon of common English words, or domain-related non-gene and non-protein terms, respectively. This figure shows some important differences between gene names of different organisms. For the comparison of organisms we focus on the results for the combined and curated dictionaries. Yeast has the lowest ambiguity with common English words as well as with domain-related terms (0.01–0.3%, resp. 0.09–0.4%). The highest degree of ambiguity with common English words was found for fly (0.55%–2.4%). This is due to frequent phenotypic descriptions that are used as gene names and abbreviations thereof (e.g. in FlyBase, *We *is the abbreviation and valid symbol for a gene named *Washed eye*, thus the abbreviation as well as the words of the long name are perfect English words). The gene nomenclature guideline for FlyBase is relatively unrestricted [29], it states that gene names must be concise, should allude to the genes function, mutant phenotype or other relevant characteristic, and gene names must be unique and not have been previously used for a *Drosophila *gene; furthermore gene names should be inoffensive. This is a relatively loose guideline, as no format is proposed for the symbols, and no restrictions about ambiguities with English words or other terms are made. The guideline rather favors the usage of descriptive names, which might be useful for an immediate functional classification of genes by a researcher when reading scientific articles, but clearly brings about significant disadvantages for literature search and automatic text processing.

The degree of ambiguity with the domain-related lexicon is also highly variable (between <0.1% and 1%). The degree of ambiguity with the lexicon of common English words agrees with the results reported for previous studies [29,30], even though the respective authors worked with a different lexicon of English words (the Moby lexicon project [31]). The degree of ambiguity with UMLS-terms was estimated to be significantly higher (7–28% for fly, human, mouse, and rat) by Chen *et al*. [30]. This might be due to their expansion of the set of UMLS terms by adding abbreviations extracted from UMLS.

Generally, the percentages of ambiguity may seem rather small, but e.g. the 2.4% of fly synonyms in the combined list ambiguous to common English words correspond to a total number of 2208 synonyms, and as several of the corresponding synonyms resemble English words that are frequently mentioned in scientific articles (e.g. *We*, *gel*, *fold*, *inactive*), they have a significant impact on any manual or automatic literature search. Furthermore, we only detected gene names that directly match entries of a lexicon. Gene names may not match to an individual entry of a lexicon, but to a combination of several entries, e.g. the gene names *Washed eye *and *legless *were not found as such in the used English lexicon, but represent combinations of usual English words, that are present in the lexicon individually. Whether such gene names are critical for detection in texts cannot be decided in general, as this depends on whether they correspond to a combination that fulfills standard English syntactic rules and on the (approximate) matching scheme employed. For some of these gene names, additional methods allow safe detection, e.g. the word *legless *can only represent a gene name when being used as noun and a phenotypic description when tagged as adjective.

### Relevance of ambiguities for mining MEDLINE

For text-mining, it is especially important to know, besides the degree of ambiguity of synonom dictionaries, the number of ambiguities that occur when mining MEDLINE. Thus, it is important to know whether ambiguous synonyms occur frequently in abstracts as this indicates the relevance of disambiguation approaches. The hypothesis 'one discourse → one meaning' reflects that, generally, semantically ambiguous terms carry the same meaning throughout a limited unit of text (e.g. the word 'bank' will rarely describe a financial institute and a seating within the same article). Here, we investigate inter-organism ambiguity. In accordance to the above assumption, we assume that synonyms which are ambiguous for different organisms and occur within a same abstract share the same context and thus refer to the same organism.

We consider all pair-wise combinations of the organisms mouse, rat, and human, and match the respective synonym dictionaries against MEDLINE abstracts. As we assume that each abstract that contains ambiguous synonyms deals with only one organism the disambiguation task is reduced to selecting for each abstract one of the two organisms under consideration. For giving a rough estimate of the relevance of ambiguities for mining MEDLINE, we apply a simple disambiguation strategy: Given an abstract that contains ambiguous synonyms and additionally contains synonyms assigned to only one of the respective organisms or mentions one of the organisms under consideration directly, all ambiguous synonyms within this abstract are assigned uniquely to this organism.

Table 2 shows the results of this relevance analysis. Out of approx. 7 million abstracts, 2.2–2.8 million abstracts were found to contain at least one gene/protein name of the considered pairs of organisms. Approximately 58–65% of the latter contain protein names that are ambiguous between the two synonym lists. 23–27% of the abstracts contain, besides at least one ambiguous synonym, synonym(s) assigned to only one of the considered lists.

17–38% of the abstracts contain, besides the ambiguous synonym(s) a unique organism name identifying one of the organisms under investigation. Finally, between 34% and 52% of the abstracts contain either a gene/protein name or an organism name unique to one of the considered organisms. Therefore, the basic organism disambiguation strategy consisting in assigning the organism that is indicated by direct mention of the organism or unique gene/protein name(s) can cover at most this percentage of abstracts. Consequently, the remaining 12.4% (human-rat) to 24.5% (mouse-rat) of the abstracts containing a gene name contain neither unique synonyms nor organism names that could easily be used for disambiguation. Thus, this fraction of abstracts containing a gene name cannot be disambiguated by the described simple strategy and thus definitively require for other disambiguation methods.

Clearly, these numbers can only represent a rough estimate as several assumptions were made for this analysis, e.g. MEDLINE abstracts do not necessarily refer to only one organism, and a mention of an organism does not necessarily imply that the gene names refer to this organism. Here, we do not evaluate quality of disambiguation by the described disambiguation approach which would imply comparison against a benchmark data set.

Yet, our results show that inter-species ambiguity is not only a problem inherent to synonym dictionaries but also directly affects text-mining of MEDLINE abstracts; ambiguous synonyms occur often in abstracts, inter-species disambiguation is not trivial, and thus more involved disambiguation approaches are definitively required.

### Use of gene name dictionaries

In summary, the curated version of the combined lists show the largest size (Figure 1) and the lowest ambiguity among dictionaries (Figure 2), English terms, and non-gene terms (Figure 4). As mentioned above, the synonym dictionaries derived in this work could be of use for numerous applications. Therefore, we provide the derived synonyms via the web. The obvious problem is to maintain those lists in order to make available current, comprehensive and curated versions. This implies that the lists need to be updated on a regular basis in order to integrate new databases and database updates. Our solution to these problems is to make the data available in a wiki. The wiki allows for regular updates. Registered users may simultaneously edit and comment entries via a convenient and easy to use interface. Updates and comments are immediately publicly available. Every object of a synonym dictionary corresponds to an entry in the wiki. Every entry contains links to the source databases as well as a query facility that allows to query MEDLINE via PubMed and Google with all synonyms of a gene/protein simultaneously.

We integrate new data periodically via the automated procedures described in this paper. The procedure for synonym dictionary generation and curation is adapted to account for changes in the underlying data sources and mappings between data sources. In addition, during such an update we systematically check the modifications made on the data and decide whether the suggestions made by the users are kept or removed from the new updated version. If necessary, we discuss suggestions with the respective registered users. This way, we hope to continuously improve the underlying curated dictionaries and make available the most current ones to the community.

## Conclusion

The results presented above clearly indicate that, when aiming at construction of a broad coverage gene name dictionary, it is important to combine information from different data sources. Such a gene name dictionary is relevant for several scenarios:

(1) The researcher, who is starting to work on a gene/protein so far unknown to him should consider several databases for obtaining information. Especially, he should use all available gene names when doing literature search. Otherwise he risks missing information on the gene/protein, e.g. previously published results on certain experiments, which may lead him to inappropriate planning of studies.

(2) Automated approaches like systems for information retrieval or information extraction that focus on genes and/or proteins benefit from comprehensive and specific input data. A gene name dictionary allows to create specific queries, integrate information on individual genes/proteins, and also to directly link information from several information sources (database mapping).

(3) Approaches for named entity recognition and identification frequently apply gene name dictionaries. Depending on the recognition methodology, they depend more or less on the dictionary, but generally they produce better results with better dictionaries. Our results in BioCreAtIvE have shown a significant influence of the dictionary on recall and precision of gene name identification when applying a simple exact text matching approach [8], but also a significant influence when applying a more involved search strategy [9].

This study shows that the ambiguity within gene names and also between gene names and common English words and domain-related terms is significant. The degree of ambiguity varies between different organisms. Some of the aspects that we analyzed here have been previously analyzed in a slightly different way (e.g. different thesaurus of common English words, different data sources for the generation of gene name dictionary) [29,30]. The results are similar and thus show a good agreement of the studies.

The ambiguity is in part due to the rules of gene and protein nomenclature for the corresponding model organisms. It is evident that a descriptive and free nomenclature as it is used for *Drosophila *makes automated identification of gene names very difficult, while a stringent nomenclature as it is used for yeast allows for easy means of gene name identification.

The ambiguity between different gene name dictionaries partially reflects the degree of relatedness of the corresponding organisms and a number of the ambiguous synonyms refer to genes that are homolog or structurally/functionally similar. The combination of knowledge from orthology maps with relationships derived from ambiguity analysis might thus provide a further means for integrating knowledge on genes and also for generating broad coverage gene name dictionaries.

The problem of how to handle synonyms ambiguous for different organisms within a named entity recognition system cannot generally be solved as a solution depends on the task at hand. When the aim is to get information for certain individual proteins within a given organism, the system should be able to distinguish between the objects of different organisms assigned to ambiguous synonyms. When it becomes important to integrate information on a higher level, e.g. to find genes relevant for a certain human disease, it might be preferable to also integrate ortholog genes or transgenes from model organisms. In this case, it would be preferable not to exclude objects from other organisms. For this application it would also be useful to ensure orthology between the corresponding objects.

The ambiguity with non-gene terms is harmful in all applications and therefore it is important to reduce this effect, especially in automated named entity recognition systems. Different strategies can be applied for this task, e.g. the reduction of ambiguities in the dictionary by an automated curation, or a strategy of ambiguity resolution during named entity recognition. The degree of ambiguity depends on the considered organism, and the attempted solution should cover the problematic cases of the specific organism. Certainly, it would be desirable to get researchers to use gene and protein names that correspond to stringent nomenclature rules, as this would facilitate every kind of literature search and automatic text processing. Nevertheless, this would not remove the difficulty entirely, as the already published literature also needs to be integrated and analyzed. Therefore, it would be highly desirable to have systems at hand that can handle the ambiguity problem in a general way and irrespectively of the organism under consideration.

Furthermore, this study shows that the degree of overlap between different public data sources is generally relatively modest. Thus, it is very important to combine information from several data sources when aiming at the construction of a broad coverage gene name dictionary. The higher parsing effort results in a significant increase in dictionary size, which allows for increased coverage.

The individual nomenclature committees and organizations were rather separated from each other when they started gathering information and first set up databases for making information available to the public. Yet, in the last years, they tend to increasingly coordinate and integrate their work by agreeing common nomenclature guidelines, exchanging data and enforcing co-assignment and co-curation of gene and protein annotation. As a consequence, the number of cross-links between databases has already significantly increased. With the advent of integrative projects like e.g. the UniProt project, the overlap of data from various databases is expected to increase significantly.

This study provides background knowledge on the size, ambiguity and overlap of gene name dictionaries derived from different public data sources and measures for quantifying these parameters. The presented results provide hints for the construction of gene name dictionaries. The proposed measures are helpful for judging the quality of gene name dictionaries and estimating their performance in named entity identification applications.

The entries of the curated synonym lists derived for this study will regularly be updated and provided to the public via a wiki [16], which also contains links to the source databases, and the possibility to query PubMed and Google for objects via the list of all synonyms. The wiki allows for browsing the lists, but also for commenting and editing their contents in a quite flexible way. This can easily be done by many users simultaneously over the web. We periodically check the modifications made by users and decide on whether to remove or keep the suggested edits. Of course, the actual content relies on the cooperation of many users and editors.

## Methods

### Compilation of gene name dictionaries

A **gene name dictionary ***d *consists of a set of objects *objects*(*d*) or *O*^*d *^representing genes or proteins, where each object *o *∈ *O*^*d *^is associated with a set of identifiers from one or more databases and a set of synonyms (gene names) *S*(*o*). The set of all synonyms **synonyms(d) **of a dictionary d is:



Gene name dictionaries were compiled for the organisms yeast, fly, mouse, rat and human. They were derived from organism-specific and general databases. As organism-specific databases, we used FlyBase for *Drosophila melanogaster *(Fly), HUGO for *Homo sapiens *(Human), Mouse Genome Informatics (MGD) for *Mus musculus *(Mouse), Rat Genome Database (RGD) for *Rattus norvegicus *(Rat), and Saccharomyces Genome Database (SGD) for *Saccharomyces cerevisiae *(Yeast). As general databases we used Swiss-Prot and Entrez Gene (the successor of the LocusLink database). The corresponding files FBgn.acode for Fly-Base, nomeids.txt and ens4.txt from HUGO, MRK_LocusLink.rpd and MRK_SwissProt_TrEMBL.rpt from MGI, GENES from RGD, registry.genenames.tab and dbxref.tab for SGD, gene_info from Entrez Gene, uniprot_sprot.dat from Swiss-Prot were downloaded from the ftp sites in June 2005.

We extracted all entries from the organism specific databases; for fly, we extracted only entries being indicated as belonging to *Drosophila melanogaster *and did not use genes from all other species of *Drosophilidae*, which are also contained in FlyBase. From Swiss-Prot and Entrez Gene, we extracted all entries corresponding to the respective organism. From Entrez Gene, only entries of type "protein-coding" were considered. Gene identifier, symbols, aliases and long names were extracted from all databases and converted into a uniform format. The extracted dictionaries contain a set of entries where each database identifier is associated with all corresponding symbols, aliases, and names. After extraction, all symbols, aliases, and names are treated equivalently, as synonyms for the object in question.

Mappings between database identifiers of the different databases were also extracted from the downloaded files. These mappings were used for generating the combined dictionaries. No transitive mappings were performed, entries were only merged if the corresponding identifiers were directly mapped to each other in one of the considered databases. For each organism under consideration, we generated a combined dictionary by joining the entries from the corresponding organism specific database and the general databases Swiss-Prot and Entrez Gene. Entries from different databases were merged into a single entry when the corresponding identifiers were mapped to each other in any of the three databases.

Finally, each of the combined dictionaries is submitted to an automated curation procedure described earlier [8,10]. This procedure aims at adding missing synonyms and removing inappropriate synonyms. The expansion is achieved mainly by adding spelling variants (e.g. a ↔ alpha, 1 ↔ I) and expansion of abbreviations that are part of a full name (e.g. IL ↔ Interleukin). Pruning is based on token-class based regular expressions. A token can be any sequence of letters and/or numbers. We define token classes as groups of words which have a similar meaning or usage. Examples of token classes are: measuring units (contains: kDa, Da, mg,...), common words (if, and, as, for, ...), descriptions (tRNA, Ser, Tyr,...), numbers, single letters. These token classes are combined in regular expressions, e.g. 'a number followed by a measuring unit', 'one description', 'a common word followed by a number'. Synonyms that match exactly one of these regular expressions are removed, e.g. '22 kDa' is removed by the regular expression 'a number followed by a measuring unit'. The lists of words belonging to a token class and the rules for combining them in regular expressions were compiled during previous work (based on analysis of synonyms provided in public databases and their matching statistics against MEDLINE abstracts). Synonyms that are equal to standard English words or are contained in a short manually defined list of inappropriate synonyms are also pruned. Objects that have no synonym left are removed from the dictionary; this concerns objects that have only unspecific synonyms like ORF-number and EC-number. Furthermore, for the unification of the synonym dictionaries used for the present study, two entries that have at least 60% of the smaller number of their synonyms in common are merged into a single entry.

This yields 25 gene name dictionaries in total; for each of the five organisms we generated three individual dictionaries (organism specific database, Entrez Gene, Swiss-Prot), one combined and one curated dictionary.

### Lexicon of common English words and domain-related non-gene or -protein terms

We used words from the Wall Street Journal (WSJ) and Brown corpus as lexicon of common English words. The lexicon as provided with Brill's part of speech tagger [32] contains 93 694 entries.

The lexicon for domain-related non-gene and non-protein terms was obtained from the Unified Medical Language System (UMLS, Version 2004AB) [33]. All terms, that do not have a semantic type related to genes or proteins ("Amino Acid, Peptide, or Protein", "Enzyme", "Amino Acid Sequence", "Gene or Genome", "Receptor", or "Hormone") assigned, were extracted from the MRCONSO.RRF file. This yields a lexicon of 1 062 223 unique entries.

### Size, degree of ambiguity and overlap

Gene symbols and long names show quite variable properties; while the case of a short name can be decisive for whether it refers to one gene or another, the spelling of long names is usually much more flexible. Therefore we apply different definitions of **equivalence **~_*e*_:

• *exact*: Two names *s*, *s*' are equivalent if they match each other in a case sensitive way: *s *~_*exact *_*s*'

• *mixed*: Two names are equivalent if they match each other, and the match is case sensitive if the name consists solely of letters and is of length less than six and case insensitive otherwise: *s *~_*mixed *_*s*'

• *normalized*: Two names are equivalent if they match each other in a case insensitive way and after any sequence of non-alphanumerical characters has been replaced by a single placeholder: *s *~_*normalized *_*s*'

The **size **of a gene name dictionary is estimated by the number of objects, i.e. distinct genes, in the dictionary (*#*(*objects*(*d*))) and the number of distinct synonyms according to the equivalence *e *(= *#*_*e*_(*x*), the number of equivalence classes in x):

*Cov*(*d*) = (*#*(*objects*(*d*)), *#*_*e*_(*synonyms*(*d*)))

A synonym *s *is said to be **ambiguous **if it is equivalent (according to the definition above) to a second synonym *s*' of a different object:

∃*s *∈ *S*(*o*) ∃*s*' ∈ *S*(*o*'): *s *~_*e *_*s*' ∧ *o *≠ *o*'

or if a synonym is equivalent to an entry *l *of a lexicon *L *of common (non-gene/non-protein) terms:

∃*s *∈ *S*(*o*) ∃*l *∈ *L*: *s *~_*e *_*l*.

The **degree of ambiguity **is the quotient of the number of ambiguous synonyms in a set *X *and the number of total synonyms in *X*.



We consider three different variants of the degree of ambiguity:

(1) Intra-dictionary degree of ambiguity: The *DoA *of a dictionary *d *is given by setting *X *= *synonyms*(*d*), i.e. *X *is the set of all synonyms for all objects in *d*.

(2) Inter-dictionary degree of ambiguity: The *DoA *for two dictionaries is computed by setting X to the set of all synonyms in both dictionaries, *ambiguous*(*X*) refers to synonyms that are ambiguous between the two dictionaries.

(3) Dictionary-lexicon degree of ambiguity: The *DoA *for a dictionary *d *and a lexicon *l *is the fraction of ambiguities between *d *and *l *and the number of all synonyms in dictionary *d *(here, the denominator does not consider the number of synonyms in the lexicon).

The **degree of overlap **of two dictionaries *d*, *d*' is determined as follows: For each organism, mappings between database identifiers are obtained from the three relevant databases (Swiss-Prot, Entrez Gene, and organism-specific). All direct mappings between database identifiers are used to generate pairs of objects (*o*, *o*') from the two dictionaries under consideration. Objects that cannot be mapped to an object of the second dictionary are ignored. For each pair of objects, the number of equivalent synonyms is determined. The fraction of the total number of equivalent synonyms to the total number of distinct synonyms belonging to the considered objects indicates the degree of overlap.



### Relevance of ambiguities for mining MEDLINE

The relevance of inter-species ambiguities for mining MEDLINE abstracts is estimated as follows: We consider all pair-wise combinations of the organisms human, mouse, and rat. The curated gene/protein name dictionaries are matched against approx. 7 million MEDLINE abstracts (from 1990 or later) by ProMiner [9,10], a system that searches gene and protein names by approximate string matching based on a token-class model and yields matches with high sensitivity and specificity (81–84% and 81–97%, respectively, for mouse, fly, yeast, as evaluated in the BioCreAtIvE challenge). This returns the number of abstracts in which a gene name was found. We then determine the number of abstracts that contain synonyms that are ambiguous between the respective pair of organisms. This indicates how often inter-species ambiguities occur when mining MEDLINE abstracts.

Assuming that abstracts (mostly) deal with single organisms, a simple disambiguation strategy is applied for each abstract containing synonyms that are ambiguous for the two considered organisms: If the abstract contains, besides the ambiguous synonym(s), further synonyms that are assigned to only one of the considered organisms, the ambiguous synonyms are also assumed to refer to this organism. For example, 'BNIP3H' is a synonym of the human gene 'BCL2/adenovirus E1B 19-kDa protein-interacting protein 3-like' (BNI3L_HUMAN) in Swissprot, but not of the corresponding gene in mouse (BNI3L_MOUSE); thus, if 'BNIP3H' occurs in an abstract, one can hypothesize that the article deals with human and thus presumably all ambiguous synonyms within this abstract also refer to human. If an abstract contains, besides ambiguous synonym(s), only one of the corresponding organism names, the ambiguous synonyms are assumed to refer to this organism. As organism names also have synonyms (e.g. human, *Homo sapiens*, *H. sapiens*), an organism name dictionary was compiled from UMLS and matched against abstracts according to the mixed equivalence described above.

Finally, the total number of abstracts that could be disambiguated by this strategy is estimated by the number of abstracts that contain, besides the ambiguous synonyms, either unique synonyms, or a corresponding organism name. Accordingly, the number of abstracts that cannot be disambiguated by this approach is determined as the difference of the number of abstracts that contain ambiguous synonyms and the number of abstracts that additionally contain either unique synonyms or a corresponding organism name. The numbers are expressed as fractions of the total number of abstracts containing a gene/protein name of the respective pair of organisms.

## Authors' contributions

KF participated in the projects design, carried out programming and analysis of results and drafted the manuscript, RZ conceived the study, participated in its design and drafted the manuscript.

## Figures and Tables

**Table 1:** Inter-species ambiguity: Degree of ambiguity between gene name dictionaries of different organisms. The three numbers in each field correspond to the three definitions of equivalence: exact, mixed, and normalized, respectively, from left to right; numbers are percentages. The upper part of the table contains values for the combined dictionaries, the lower part the values for the curated dictionaries (combined dictionaries are compared against combined dictionaries, and curated against curated dictionaries). For details see section 'Inter-species ambiguity'.

	Human	Mouse	Rat	Yeast
Fly	1.4/1.9/2.4	1.6/1.9/2.3	1.1/1.4/1.7	0.9/1.3/1.4
Human		15.1/22.5/24.8	8.5/12.8/14.3	2.3/2.5/2.5
Mouse			13.5/13.9/14.1	1.2/2.0/2.1
Rat				1.0/1.7/1.8

Fly	0.9/1.6/1.9	1.0/1.6/1.8	0.8/1.3/1.5	0.5/1.0/1.1
Human		13.6/24.8/25.5	9.5/16.4/17.3	1.9/2.1/2.1
Mouse			17.4/18.7/18.0	0.9/1.8/1.7
Rat				0.6/1.4/1.4

**Table 2:** Relevance of inter-dictionary ambiguities for mining MEDLINE (amb.: ambiguous). The column 'nb. found abstracts' contains the number of MEDLINE abstracts (from within a set of approx. 7 million abstracts) that contain at least one gene/protein name of the respective organisms. The values in the other columns are percentages of the values in the column 'nb. found abstracts'.

	nb. found abstracts	% amb. abstracts	% amb.+ unique synonym	% amb.+ unique organism	% amb.+ unique synonym or organism
human-mouse	2 761 987	60.5	23.1	37.8	46.5
human-rat	2 238 212	64.5	27.2	43.5	52.1
mouse-rat	2 532 682	58.2	24.2	17.1	33.7
